# Susceptibilities of *Candidatus* Liberibacter asiaticus‐infected and noninfected *Diaphorina citri* to entomopathogenic fungi and their detoxification enzyme activities under different temperatures

**DOI:** 10.1002/mbo3.607

**Published:** 2018-03-25

**Authors:** Mubasher Hussain, Komivi Senyo Akutse, Yongwen Lin, Shiman Chen, Wei Huang, Jinguan Zhang, Atif Idrees, Dongliang Qiu, Liande Wang

**Affiliations:** ^1^ Plant Protection College Fujian Agriculture and Forestry University Fuzhou China; ^2^ State Key Laboratory of Ecological Pest Control for Fujian and Taiwan Crops Fujian Agriculture and Forestry University Fuzhou 350002 China; ^3^ College of Horticulture Fujian Agriculture and Forestry University Fuzhou China; ^4^ Key Laboratory of Biopesticide and Chemical Biology Ministry of Education Fuzhou China; ^5^ International Centre of Insect Ecology and Physiology Nairobi Kenya; ^6^ Institute of Beneficial Insects Fujian Agriculture and Forestry University Fuzhou China

**Keywords:** asian citrus psyllid, bacterial infection, detoxification enzymes, entomopathogenic fungi, microbial ecology, temperature effects

## Abstract

Some entomopathogenic fungi species, *Isaria fumosorosea*, and *Hirsutella citriformis* were found to be efficient against the Asian citrus psyllid, *Diaphorina citri* Kuwayama (Hemiptera: Liviidae). However, the susceptibility to these fungi increases when the psyllid infected with *Candidatus* Liberibacter asiaticus (Las), which is transmitted by *D. citri* and causes citrus greening disease. In this study, we examined the Las‐infected and Las‐uninfected *D*. *citri* susceptibility to entomopathogenic fungi at different temperature regimes (5–40°C). When *D. citri* adults exposed to cold temperature (5°C), they showed less susceptibility to entomopathogenic fungi as compared with control (27°C). Irrespective of infection with Las, a significantly positive correlation was observed between temperature and percentage mortality caused by different isolates of *I. fumosorosea*, 3A *Ifr*, 5F *Ifr*, PS *Ifr*, and *H. citriformis* isolates, HC3D and 2H. In contrast, a significantly negative correlation was found between temperature and percentage mortality for 3A *Ifr* for both Las‐infected and Las‐uninfected psyllids. Detoxification enzymes, Glutathione S‐transferase levels in *D*. *citri* showed a negative correlation, whereas cytochrome P450 and general esterase levels were not correlated with changes in temperature. These findings revealed that detoxification enzymes and general esterase levels are not correlated with altered susceptibility to entomopathogenic fungi at the different temperature regimes. Conclusively, temperature fluctuations tested appear to be a significant factor impacting the management strategies of *D. citri* using entomopathogenic fungi.

## 1 INTRODUCTION

The Asian citrus psyllid (ACP), *Diaphorina citri *(Hemiptera: Liviidae), is native to Asia and southeastern Florida and has invaded several regions of the world (Capoor & Viswanath, 1967; Grafton‐Cardwell, Stelinski, & Stansly, 2013; Huang, Tsai, & Wang, 1984; Nava et al., 2010; Yan, Zeng, & Zhong, 2015). *Diaphorina citri* primarily attacks young flush of citrus trees but can also attack stressed citrus trees if the pest population density is high. It has become the most important insect pest of citrus in southeastern Florida (Halbert, 1997) and has recently threatened native citrus plants in China (Yan et al., 2015). *Diaphorina citri* greatly reduces the production, destroys the economic value of the fruits and eventually kills citrus trees when inoculates healthy citrus plants with phloem‐limited bacteria (*Candidatus Liberibacter* spp.) that cause citrus greening disease (huanglongbing = HLB) (Hall & Rohrig, 2015).

Currently, many biocontrol agents have been used against *D. citri* (Khan, Arif, Hoddle, & Hoddle, 2014; Qureshi, Rogers, Hall, & Stansly, 2009) but the most effective agents are entomopathogenic fungi, *Isaria fumosorosea* Wize (= *Paecilomyces fumosoroseus*) (Hypocreales: Cordycipitaceae) and *Hirsutella citriformis* Spear (Hypocreales: Ophiocordycipitaceae) (Avery et al., 2013; Speare 1920). These biocontrol agents have received interest for use in the management of *D. citri* in Florida (Avery et al., 2011; Hall et al., 2012). During summer, *D. citri* adults disperse, and females lay eggs on suitable citrus trees (Capoor & Viswanath, 1967; Lewis‐Rosenblum, Martini, Tiwari, & Stelinski, 2015; Tsai & Liu, 2000). *Diaphorina citri* is under good biocontrol program in warmer locations on citrus trees (Dahlsten et al., 1998). Entomopathogenic fungi then applied to these citrus trees, where they infect *D. citri* nymphs and adults (Avery et al., 2013).

However, the susceptibility of insects and mites to pesticides has changed due to some abiotic factors such as temperature, rainfall, and humidity, as well as nonenvironmental factors including pesticide coverage, host plants, and host infection status (Grafton‐Cardwell et al., 2013; Musser & Shelton, 2005; Tiwari, Mann, Rogers, & Stelinski, 2011; Xie et al., 2011; Yang, Margolies, Zhu, & Buschman, 2001). Insecticides toxicity depends on the target pest and application method at given temperature (Musser & Shelton, 2005). In tropical and sub‐tropical areas where *D. citri* are present, the variation in toxicity of insecticides is caused by different temperature variations ranging between 5°C and 40°C (Boina, Onagbola, Salyani, & Stelinski, 2009). In some areas of the world, like Florida *D. citri* are occasionally exposed to cold stress below −6.5°C and −8°C; and it was reported that *D. citri* adults and nymphs tolerate these cold temperatures (Hall, Wenninger, & Hentz, 2011; Hussain, Lin, & Wang, 2017; Hussain, Akutse, et al., 2017). Thus, lethal concentration (LC_50_) values of chemical insecticides for *D. citri*, based on the temperature regimes have been previously investigated (Boina et al., 2009). However, this lethal effect as regards temperatures has not been studied with entomopathogenic fungi. Additionally, the effect of unfavorable temperature stress on entomopathogenic fungi pathogenicity has not been investigated for *D. citri*.

In this study, susceptibility of *D. citri* to entomopathogenic fungi under cold stress (5°C) was investigated and compared with control (27°C). Furthermore, experiments were conducted to assess Las‐infected and Las‐uninfected *D. citri* detoxification enzymes activity levels; glutathione S‐transferase (GST), cytochrome P450, and general esterase, at different temperature regimes. Tiwari et al. (2011) have previously reported that these detoxifying enzyme systems were correlated with *D. citri* insecticide resistance. The aim of this study was also to investigate the effect of different entomopathogenic fungi, temperature and pathogenicity correlations in Las‐infected and Las‐uninfected psyllids.

## 2 MATERIALS AND METHODS

The original population of *D. citri* was initially collected from Fuzhou (FzP, 26.07877° N, 119.2969° E), Fujian China from *Murraya paniculata* (L.) Jacq. (Sapindales: Rutaceae) Plants (Orange Jasmine). The stock populations were maintained for about nine generations prior to experiments on the same host plants kept in mesh cages (50 × 50 × 50 cm) in greenhouse at a temperature of 27 ± 1°C, a photoperiod of 14:10 light/dark (L/D; 14 hr light 6:00–20:00), and 75 ± 5% relative humidity (RH), with no insecticide exposure. To obtain clean and homogenous colony of Las‐infected and uninfected psyllids colonies for the experiments, the apparently clean above stock populations were transferred onto Orange jasmine plants grown from seeds in an insect‐proof greenhouse at 28°C, 40% RH and L16: D8 for 4 months in mesh cages (50 × 50 × 50 cm). The 4‐month‐old seedlings were infected by grafting of Orange jasmine with four pieces of bud wood sticks from a PCR‐positive HLB source. The infection was determined and confirmed using PCR as described by Tatineni et al. (2008). Infected and uninfected orange jasmine were used in this rearing of the psyllids, which were also tested by real‐time PCR for the presence or absence of Las infection before the bioassays (Lin et al., 2016). Before each experiment, the purity of cultures HLB‐free or HLB‐infected established colonies was further checked regularly by the random amplified polymorphic DNA polymerase chain reaction (RAPD‐PCR) technique and further tested with mtCOI sequencing as described by Tatineni et al. (2008) for infection (established colony).

In this study, five entomopathogenic fungal isolates from two genera; *Isaria fumosorosea* isolates 3A *Ifr*, 5F *Ifr*, PS *Ifr*, and *Hirsutella citriformis* isolates HC3D and 2H were used against *D. citri*. The fungal isolates were cultured on potato dextrose agar (PDA) plates and were maintained at 25 ± 2°C in complete darkness. For bioassay, entomopathogenic fungi conidia were scraped off from 2‐week‐old plates with a sterilized spatula and suspended in 20 ml of autoclaved deionized water containing 0.03% Tween 80. The conidial suspensions were vortexed in for 5 min to produce homogenous conidial suspensions and then filtered through Miracloth. Fungal conidia were counted using a Neubauer Hemacytometer, and concentrations were determined through dilution. The required conidial suspension with a standard concentration of 1 × 10^8^ conidia/ml was obtained for the five entomopathogenic fungal isolates by serial dilutions containing 0.03% Tween 80 (Fluka) as a wetting agent.

### 2.1 *Diaphorina citri* susceptibility to entomopathogenic fungi at cold temperature

Prior to experiments, psyllids were carefully collected from the above tested pure (HLB‐free) and HLB‐infected colonies of *D. citri* and transferred onto clean citrus plants in mesh cages (50 × 50 × 50 cm). The mesh cages containing *D. citri* were shifted to climate box set at 5°C, 55% RH, and at a photoperiod of 14:10 hr L:D or were maintained at 27°C (control) for 1 or 2 weeks before the experiment. Psyllids from 5°C temperature were assessed using a leaf‐dip Petri dish method described by Kumar, Poehling, & Borgemeister (2005)) and Tiwari et al. (2011). Plastic disposable Petri dishes consisted of 60‐mm diameter and 1.5% agar solution (2 mm solidified layer) is used for bioassay. Leaf disks from fresh citrus leaves were cut (60 mm), dipped for 1–3 min in entomopathogenic fungal conidial suspensions prepared in 0.03% Tween 80 as described above, and left for air dry under a hood for 1 hr before bioassays. Leaf disks were dipped in 0.03% Tween 80, as the control treatment. Leaf disks (Grafton‐Cardwell et al., 2013; Yan et al., 2015) were placed on agar layers after 1 hr, and 20–30 adult psyllids of mixed gender were shifted to the dish using a soft camel hair brush. Psyllids were anesthetized shortly (10 sec) with cold temperature for easy handling and transfer. Petri dishes were sealed with parafilm (Laboratory film, PM‐996, USA) to block the psyllids. Sealed Petri dishes with adult insects were transferred into a growth chamber (Safe, China) set at 26 ± 1°C, 55% RH, and at a photoperiod of 14:10 hr L:D. Psyllids mortality was recorded 48 hr after placing the Petri dishes into the growth chamber. Psyllids were considered dead when seemed their sides or backs and not able to move when touched with a soft camel hair brush. Each fungal isolate was replicated three times, and all bioassays were repeated three times at each temperature over time. In addition, mycosis test was also conducted with the cadavers to assess fungal growth and confirm if the mortality is due to the fungal isolates infection in the various treatments.

### 2.2 Effect of temperature on enzymes levels

The effect of temperature on three detoxifying enzymes expression levels was studied by using the Las‐uninfected colony. Treatments consisted of 5F *Ifr,* or HC3D‐treated adults maintained for 48 hr at six different temperature regimes (5, 10, 20, 27, 35, and 40°C). Each entomopathogenic fungal isolate was repeated three times at each temperature, and for each replication 80–100 adult psyllids were tested. Psyllids of mixed gender were transferred onto leaves sprayed with entomopathogenic fungal isolate suspensions at the concentration of 1 × 10^8^ conidia/ml, using the Petri dish method as described above. About 80–100 adult psyllids were shifted to each Petri dish. Psyllids which survived from each treatment after 48 hr exposure were collected and used immediately for detoxifying enzymes expression levels assays.

The enzymes were prepared by following previously described methods (Gao & Zhu, 2000; Smith et al., 1985; Zhu & Gao, 1999) and then recording the absorbance at 490 nm with a 96‐well plate reader (ELISA plates, FEP‐100‐096, JET BIOFIL, China) at 25 ± 1°C. General esterase activity was calculated using α‐naphthyl acetate (α‐NA) (Sigma‐Aldrich, China) as a substrate (Tiwari et al., 2011). GST activity was calculated using 1‐chloro‐2,4‐dinitrobenzene (CDNB) (Sigma‐Aldrich) (Habig, Pabst, & Jakoby, 1974; Tiwari, Pelz‐Stelinski, Mann, & Stelinski, 2011) as substrate. Cytochrome P450 activity was measured by calculating heme peroxidase activity (Tiwari et al., 2011; William & Janet, 1997). As heme consists of cytochrome P450 in nonblood feeding arthropods, the quantification of heme activity can be used in comparing the levels of cytochrome P450 (Casimiro, Coleman, Hemingway, & Sharp, 2006; Penilla et al., 2007; William & Janet, 1997). Heme peroxidase activity was calculated by using 3,3,5,5‐tetramethylbenzidine (TMBZ) substrate (Sigma‐Aldrich).

### 2.3 Susceptibility of Las‐infected and Las‐uninfected *Diaphorina citri* to entomopathogenic fungi under different temperatures

Entomopathogenic fungi bioassays were conducted using a leaf‐dip Petri dish method as described above. Petri dishes containing the sprayed leaves at 1 × 10^8^ conidia/ml of conidial suspensions of each fungal isolate and adult psyllids were wrapped with parafilm and transferred into temperature‐controlled growth chambers set at different temperatures: 5, 10, 20, 27, 35, or 40°C with 55 ± 5% RH and 14:10 hr L:D photoperiod. For all entomopathogenic fungal isolates, treatments (Las‐infected and uninfected *D*. *citri*) were replicated three times, where each replicate comprised of *n *= 80–128 psyllids. Each entomopathogenic fungal treatment and the control were replicated three times, and the whole bioassay was repeated twice. Psyllid mortality was calculated 48 hr after shifting to the growth chamber. Psyllids were considered dead when seemed their sides or backs and not able to move when touched with a soft camel hair brush.

For bioassays using Las‐infected psyllids, each live or dead *D*. *citri* was transferred into a sterile 2 ml microcentrifuge tube (Promega, China) consisting of 80% ethanol, and kept at −20°C for further analysis. After mortality data were calculated, DNA was again extracted from the exposed Las‐infected psyllids, just for reconfirming Las infection by using quantitative real‐time PCR following the previously described protocol (Tiwari, Lewis‐Rosenblum, Pelz‐Stelinski, & Stelinski, 2010). Mortality data obtained from the two bioassays conducted with psyllids were collected for subsequent analyses.

### 2.4 Statistical analysis

The mean mortality (%) among psyllids exposed to various entomopathogenic fungi was analyzed using Analysis of Variance (ANOVA) test, and correlation analyses were conducted at *p* < .05, between Las‐infected and Las‐uninfected psyllid treatments, entomopathogenic fungi, and temperature regimes. Las‐infected and Las‐uninfected mean mortalities for the various entomopathogenic fungi and at the different temperatures were compared using Chi‐square. Mortality percentages in all treatment were corrected using Abbott's formula (Abbott, 1925). To calculate the effects of temperature and entomopathogenic fungi on mean mortality (%) of psyllids, ANOVA tests and Fisher's LSD mean separation tests were performed. The effect of temperature on detoxifying enzyme levels was measured individually for each entomopathogenic fungi by ANOVA test (*p *< .05), whereas the correlation analyses between detoxifying enzymes expression levels and temperature were determined separately for each entomopathogenic fungi and enzyme combination. SPSS 19.0 statistics software was used to perform all the data analysis.

## 3 RESULTS

### 3.1 Susceptibility of *Diaphorina citri* to entomopathogenic fungi at cold temperature regime

Percentage mortality comparisons which were performed between cold temperature and control psyllids for each period and entomopathogenic fungi showed that when *D. citri* were exposed to cold stress (5°C) for 1 week, they were significantly less susceptible to 5F Ifr than control psyllids at 27°C (Table 1). Similarly, psyllids that were exposed to cold stress (5°C) for 2 weeks were less susceptible to 2H than psyllids maintained at 27°C. However, when mortalities were compared between psyllids exposed to cold temperature (5°C) and the control (27°C), no significant differences were observed for the other tested entomopathogenic fungi (3A *Ifr*, PS *Ifr*, and HC3D; Table 1).

**Table 1:** Mean mortality (%) of *Diaphorina citri* adults by entomopathogenic fungi after preexposure periods of 1 or 2 weeks at cold stress (5°C) or control temperature (27°C)

Entomopathogenic fungus	(5°C)	(27°C)	*p* value
	1‐week exposure period		
3A *Ifr*	70.0 ± 0.5	80.4 ± 4.6	.146
5F *Ifr*	56.5 ± 7.0	84.7 ± 2.4	.0001^a^
PS *Ifr*	93.1 ± 0.1	87.2 ± 1.0	.226
HC3D	78.1 ± 1.1	83.5 ± 0.4	.249
2H	81.6 ± 3.5	87.8 ± 0.4	.322
	2‐week exposure period		
3A *Ifr*	88.8 ± 2.1	80.1 ± 5.0	.144
5F *Ifr*	83.0 ± 3.4	92.1 ± 1.0	.121
PS *Ifr*	96.4 ± 1.5	91.1 ± 1.1	.824
HC3D	76.6 ± 3.0	81.3 ± 4.4	.201
2H	71.0 ± 4.2	89.1 ± 1.2	.024^a^

a Represent significant differences in mortality between entomopathogenic fungi and temperature interactions at different exposure period, at *p* < .05.

### 3.2 Effect of temperature on different enzymes levels

**Figure 1:**
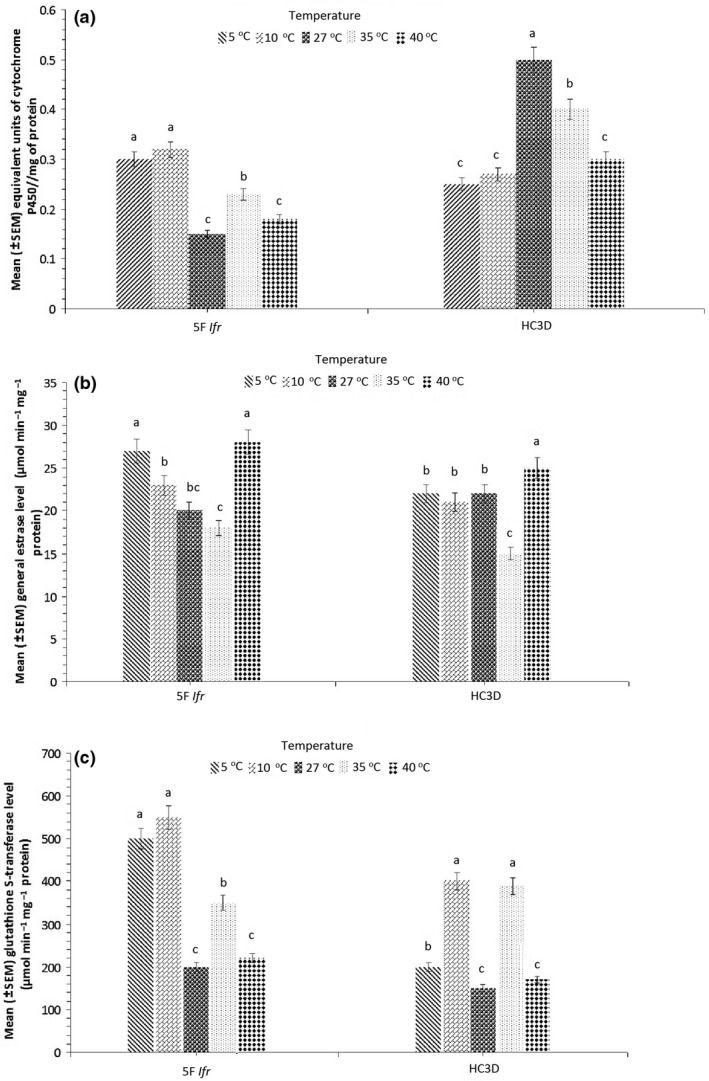
Cytochrome P450 (a), general esterase (b) and glutathione S‐transferase (c) activity levels comparison in Las‐uninfected *Diaphorina citri* at different temperatures regimes. Bars represent means with standard errors (SE), and means followed by different letters indicate a significant difference at *p* < 0.05

Psyllids treated with 5F *Ifr* (*F*_5,10_ = 1.0; *p* = .1451) or HC3D (*F*_5,10_ = 0.22; *p* = .4236) had nonsignificant effect on cytochrome P450 activity at all the temperature regimes (Figure 1a). Similarly, correlation analysis showed nonsignificant association between temperature and cytochrome P450 activity for psyllids treated with 5F *Ifr* (*r* = −0.2520, *p* = .1340) and HC3D (*r* = 0.0100, *p* = .6226). General esterase activity levels of psyllids treated with 5F *Ifr* (*F*_5,10_ = 0.43; *p* *=* .4202) or HC3D (*F*_5,10_ = 1.12; *p* = .2073) had positive effect of temperature (Figure 1b). Temperature and general esterase activity in psyllids treated with 5F *Ifr* (*r* = 0.0623, *p* = .5226) or HC3D (*r* = 0.1356, *p* = .2207) had nonsignificant relationship. However, when comparing the cytochrome P450 and general esterase activities (Figures 1a, b), temperature had significant effect on the GST activity levels for psyllids treated with 5F *Ifr* (*F*_5,10_ = 2.11; *p* = .0213) or HC3D (*F*_5,10_ = 8.11; *p* = .0034) (Figure 1c). A negative correlation was observed between temperature and GST activity for psyllids treated with 5F *Ifr* (*r* = −0.6020, *p* = .0024) or HC3D (*r* = −0.5345, *p* = .0206).

### 3.3 Susceptibility of Las‐infected and Las‐uninfected *Diaphorina citri* to entomopathogenic fungi under different temperatures

Our results showed that the interactions between psyllids treatments (Las‐infected and Las‐uninfected psyllids), temperature and entomopathogenic fungus had a significant effect on the mean mortality (%) of psyllids (Table 2). In Las‐infected psyllids, entomopathogenic fungi (*F*_4,50_ = 63.40, *p* = .0034) and temperature (*F*_5,50_ = 13.10, *p* *=* .0011) had each a significant effect on *D. citri* mortality; however, the relationship effects between these factors was nonsignificant (*F*_20,50_ *=* 0.70, *p* = .5102). Data analysis showed that the temperature regimes had significant effects on the mortality of psyllids for 3A *Ifr* (*F*_5,117_ = 12.41, *p* *=* .0021), 5F *Ifr* (*F*_5,117_ = 49.41 *p* = .0012), PS *Ifr* (*F*_5, 117_ = 3.22, *p* = .013), HC3D (*F*_5,117_ = 4.24, *p* = .0133) and 2H (*F*_5,117_ = 3.10, *p* = .001) (Table 3). Likewise, a significantly positive correlation was observed between temperature and psyllids mortality for 5F *Ifr* (*r* = 0.8131, *p* = .0001), PS *Ifr* (*r* = 0.6101, *p* = .0001), HC3D (*r* = 0.8224, *p* = .0023), and 2H (*r* = 0.6501, *p* = .0024). In contrast, there was a significantly negative correlation between temperature and psyllids mortality for 3A *Ifr* (*r* = −0.6002, *p* *=* .0001).

**Table 2:** Interaction effects of temperature and Las infection on the susceptibility of *Diaphorina citri* to entomopathogenic fungi

Factors	*df*, residuals	*F* value	*p* value
Las infection	1, 214	19.60	.0035^a^
Temperature	5, 214	13.17	.0045^a^
Entomopathogenic fungi	4, 214	10.27	.0020^a^
Las infection × temperature	5, 214	1.15	.144
Las infection × entomopathogenic fungus	4, 214	5.63	.0023^a^
Temperature × entomopathogenic fungus	20, 214	15.81	.0049^a^
Las infection × temperature × entomopathogenic fungus	20, 214	0.32	.6115

a Represent significant factors and interactions at *p* < .05.

**Table 3:** Effect of temperature on pathogenicity of various entomopathogenic fungal isolates to *Diaphorina citri*

Treatment		Mean mortality % (± *SE*)	
Entomopathogenic fungus	Temperature (°C)	Las‐infected	Las‐uninfected
3A *Ifr*	5	93.0 ± 2.1aA	85.3 ± 1.6aA
	10	85.1 ± 3.4aA	73.0 ± 1.2abA
	20	79.0 ± 2.2aA	65.0 ± 1.1abB
	27	85.2 ± 4.7aA	66.0 ± 1.0bcB
	35	55.4 ± 4.7bA	62.8 ± 4.0cdA
	40	59.3 ± 1.0bA	54.7 ± 4.2dA
5F *Ifr*	5	73.5 ± 0.3cA	50.3 ± 0.4cC
	10	74.0 ± 1.2cA	56.0 ± 8.3bcC
	20	63.0 ± 1.3cA	49.0 ± 6.1bcC
	27	84.6 ± 1.5bA	65.1 ± 6.0bcC
	35	90.0 ± 0.01aA	72.0 ± 2.0abB
	40	100.0 ± 0.0aA	87.4 ± 1.5aA
PS *Ifr*	5	72.5 ± 2.4bA	58.0 ± 1.1bC
	10	74.6 ± 8.4bA	59.1 ± 2.1bC
	20	68.5 ± 6.2bA	50.0 ± 2.0bB
	27	88.0 ± 2.0aA	65.0 ± 2.8bC
	35	91.2 ± 1.5aA	66.8 ± 4.1bC
	40	97.0 ± 0.5aA	88.8 ± 3.0aA
HC3D	5	59.8 ± 1.4cB	65.7 ± 0.6cA
	10	69.3 ± 4.7bcA	70.0 ± 3.0bcA
	20	62.2 ± 3.5bcA	60.0 ± 2.1bcA
	27	78.7 ± 3.0bcA	79.3 ± 1.0bA
	35	83.5 ± 7.0bA	79.0 ± 1.7bA
	40	99.0 ± 0.2aA	91.5 ± 1.2aA
2H	5	65.4 ± 7.3bA	52.0 ± 6.0cB
	10	68.0 ± 3.6bA	64.1 ± 2.5cA
	20	61.0 ± 2.4bA	58.0 ± 2.2cA
	27	82.8 ± 8.1abA	70.0 ± 4.1bcA
	35	93.0 ± 1.2aA	85.6 ± 3.6abA
	40	97.1 ± 1.2aA	91.1 ± 1.6aA

Means with the same lower case letters within a column of each fungal isolate at the different temperatures are not significantly different at *p* < .05. And means with the same upper case letters within rows are not significantly different.

For the Las‐uninfected psyllids, entomopathogenic fungi (*F*_5,117_ = 15.23, *p* *=* .0001) and temperature (*F*_4,117_ = 2.52, *p* = .0063) and the interaction between these factors had significant effects on *D. citri* mortality (*F*_20,117_ = 7.13, *p* = .0001). For all entomopathogenic fungi tested, temperature had a significant effect on psyllids mortality for 3A *Ifr* (*F*_5,17_ = 8.52, *p* = .0002), 5F *Ifr* (*F*_5,17_ = 9.17, *p* *=* .0002), HC3D (*F*_4,17_ = 5.51, *p* = .0002) and 2H (*F*_5,17_ = 9.12, *p* = .0001). In contrast, temperature had no significant effect for PS *Ifr* (*F*_5,17_ = 1.42, *p* = .1010) (Table 3). In addition, a significantly positive correlation was observed between temperature and psyllids morality for, 5F *Ifr* (Pearson correlation coefficient; *r* = 0.6010, *p* = .0001), PS *Ifr* (*r* = 0.3154, *p* = .0121), HC3D (*r* = 0.7112, *p* *=* .0001), and 2H (*r* = 0.8031, *p* *=* .0001). It was also observed that a significantly negative correlation exists between temperature and psyllids mortality for 3A *Ifr* (*r* = −0.9105, *p* = .0001).

The comparison of the correlation coefficients for Las‐infected and Las‐uninfected psyllids showed no significant differences among Las‐infected and uninfected psyllids for 3A *Ifr* (*z* = −0.40, *p* = .5023), 5F *Ifr* (*z* = −2.00, *p* = .202), PS *Ifr* (*z* = 0.13, *p* = .7001), HC3D (*z* = 0.23, *p* = .625) and 2H (*z* = 0.61, *p* = .220) at the various temperature regimes (Table 3).

## 4 DISCUSSION

The results of the study showed that psyllids exposed to cold stress were more tolerant to entomopathogenic fungi than controls, seems to be possible effects that could be observed during winter compared to summer temperature variations. However, the cold temperature did not affect the susceptibility of *D. citri* to PS *Ifr*, 3A *Ifr*, and HC3D. Further studies are warranted to understand the mechanism underlying decreased susceptibility of cold stress psyllids populations to entomopathogenic fungi. Some studies reported that decrease in susceptibility might influence the management of psyllids under field conditions using *I. fumosorosea* and *H. citriformis* (Lezama‐Gutiérrez et al., 2012; Pérez‐González, Sandoval‐Coronado, & Maldonado‐Blanco, 2016).

Temperature affects the rate of metabolism (Hussain, Akutse, et al., 2017; Hussain, Lin, et al., 2017), the binding of the enzyme with its substrate (Hochachka & Somero, 1984) and the rate of enzymatic activity (Hoffmann, 1984). Therefore, we assumed that the different levels of susceptibility of psyllids to entomopathogenic fungi due to temperature changes observed in this study might comprise altered levels of different enzyme activities. The data analysis showed that temperature affected the levels of GST, but not of cytochrome P450 and general esterase activity. A significant reduction in levels of GST was observed at 40°C in psyllids treated with 5F *Ifr* and HC3D. The decline in GST activity does not induce a decrease in mortality of the psyllids treated with 3A *Ifr* at 40°C. However, GST reduction would be expected to increase psyllids susceptibility to entomopathogenic fungi at high temperatures rather than decreasing susceptibility. Our results showed that, in response to temperature changes, susceptibility of psyllids to entomopathogenic fungi was not correlated with detoxifying enzyme activity. Therefore, the temperature‐related temperature changes, in the psyllid susceptibility to entomopathogenic fungi may be due to other factors. However, many studies have investigated the effect of temperature on detoxifying enzymes that are due to the effect of synthetic insecticides (Chandler, King, Jewess, & Reynolds, 1991; Hodjati & Curtis, 1999; Wadleigh, Koehler, Preisler, Patterson, & Robertson, 1991).

Our results also underlined the susceptibility correlation effects of Las‐infected and uninfected psyllids to entomopathogenic fungi as regards to the changes in temperatures, which was poorly understood previously. Boina et al. (2009) reported positive correlation between temperature and synthetic insecticide susceptibility for Las‐uninfected *D. citri*. As the detection of Las infection has increased in Asia and some other parts of the world (Bove & Ayres, 2007; Chen et al., 2010; Islam et al., 2012; Morris, Erick, & Estes, 2009; Puttamuk et al., 2014; Wang et al., 2013), the proportion of Las‐infected psyllids is in some cases more than 95% (Coy & Stelinski, 2015). However Las‐infected psyllids were more susceptible to some synthetic insecticides than Las‐uninfected psyllids (Tiwari, Pelz‐Stelinski, & Stelinski, 2011). Our results showed that fluctuation in temperature is correlated with Las‐infected and uninfected *D. citri* susceptibility to entomopathogenic fungi. A general trend in Las‐infected and uninfected *D. citri* was observed; with a positive correlation between temperature and mortality for PS *Ifr*, 5F *Ifr*, HC3D and 2H, and a negative correlation for 3A *Ifr*.

Although previous studies have been conducted to show the effect of temperature on synthetic insecticides susceptibility, the alteration in entomopathogenic fungi pathogenicity due to changes in temperature was not understood (Deng, Zhang, Wu, Yu, & Wu, 2016; Garcia et al., 2016; Lasa, Williams, & Caballero, 2008; Zhang et al., 2015). However, susceptibility to entomopathogenic fungi was increased in Las‐infected as compared to uninfected *D. citri*, but Las infection did not affect the correlation coefficients between temperature and psyllids mortality.

Recently, biopesticides are developed as the best tool for management of herbivores like *D. citri*; thus the knowledge of interactions between abiotic factors (temperature) and herbivore susceptibility to biopesticides may help to understand and design better management strategies against the pests. Our results showed that alterations in psyllids susceptibility to entomopathogenic fungi are due to changes in temperature, which are not related to changes in detoxifying enzymes expression levels. However, psyllids exposed to cold stress are less susceptible to entomopathogenic fungi *I. fumosorosea* and *H. citriformis* than control. Thus, seasonal variations in temperature and entomopathogenic fungi may have a major impact on the management of *D. citri* under field conditions.

## CONFLICT OF INTEREST

None declared.
